# Effect of cassava (*Manihot esculenta* Crantz) varieties on leaf bud sprouting for rapid multiplication of planting materials

**DOI:** 10.3389/fpls.2024.1453538

**Published:** 2025-01-17

**Authors:** Ngakwi Masai Meibuko, Hosea Dunstan Mtui, Anna Baltazari

**Affiliations:** ^1^ Tanzania Agriculture Research Institute, Tumbi Centre, Tabora, Tanzania; ^2^ Department of Crop Science and Horticulture, College of Agriculture, Sokoine University of Agriculture, Chuo-Kikuu, Morogoro, Tanzania; ^3^ Pesticides Bio-efficacy Section, Tanzania Plant Health and Pesticide Authority, Arusha, Tanzania

**Keywords:** leaf bud position, percent sprouting, survival rate, cassava mosaic disease, cassava brown streak disease, variety

## Abstract

A drawback associated with conventional clonal propagation of cassava is its limited multiplication ratio, which poses challenges for both cassava crop enhancement and seed system initiatives. This study was aimed to evaluate the response of varieties on the sprouting ability of their leaf buds from the upper, middle, and lower positions of mature plant branches for rapid multiplication of planting materials. Five varieties, namely, Kizimbani, Mkombozi, Mkumba, TARICASS 4, and Mkuranga 1, were selected. The criteria for selection were resistance to green mites (*Mononychellus tanajoa*), a common cassava serious insect pest; Cassava Mosaic Disease (CMD) and Cassava Brown Streak Disease (CBSD), the most serious cassava viral diseases; and high-yielding, dry matter, and starch content potentials. The experiment was conducted in a screen house where the leaf buds from mature mother plant branches were planted in a growth cage measuring 3.0 m × 1.5 m × 3.5 m made of metal pipes and covered with a transparent polyethylene sheet for sprouting. The design used was randomized complete block design (RCBD) in a split–split plot arrangement with three replications. Varieties were the main plot factor, media as a subplot factor, and leaf bud position as a sub–sub plot factor. Results showed that variety had significant effects (p < 0.001) on number of nodes, percent sprouting, number of sprouts, and days to 50% sprouting, and a significant difference (p < 0.01) on percent survival rates and days to first sprouting. The effects of leaf bud position had significant effects (p < 0.001) on the number of leaf buds, percent sprouting, number of sprouts, days to first sprouting, and percent survival rates. The media used showed significant effects on days to first sprouting (p < 0.01) and first sprouting. TARICASS 4 was the most promising variety for rapid multiplication of cassava planting materials using the leaf bud technique due to its superior performance. The use of coco peat and peat moss media generally led to early and more successful sprouting. These results are important as a basis for selecting varieties for propagation using the leaf bud technique for rapid multiplication of planting materials for breeding and seed production purposes.

## 1 Introduction

Cassava (*Manihot esculenta* Crantz) is an important crop in Tanzania, providing food and income for millions of people in the country (Mashenene et al., 2023). Cassava production and productivity among other factors mostly depend on good breeding and seed systems programs, and adequate availability of high quality planting materials (Kongsil et al., 2024). The most commonly used method for cassava seed multiplication is through stem cuttings (de Oliveira et al., 2020). This method is relatively simple and inexpensive, but it can take several months for the cuttings to develop into mature plants, which slows down the process of seed multiplication (Ferguson et al., 2019). On average, a mature cassava plant at 8 months after planting (MAP) can yield 5 to 10 cuttings, resulting in a ratio of 1:10 (Ceballos et al., 2004; Oketade, 2023) in contrast to crops like maize, where a single grain has the capacity to multiply up to 300 viable grains (Ferguson et al., 2019);. As a result, rapid commercial cassava expansion of production area takes a long time due to difficulties to collect sufficient planting materials limiting the need for replacement of landraces by improved varieties with superior yield and disease resistance traits (Neves et al., 2018; Sulle et al., 2022). To address the challenge, various techniques have been developed for accelerating multiplication of cassava seeds. Some of these techniques include the use of tissue culture, yet its main drawback is high cost (Okpara et al., 2022) and use of nodal cuttings (Muktar et al., 2024). Despite such efforts, still very few farmers are using improved varieties, due to unavailability of planting materials from the released varieties (Murray and Cohen, 2021). Reducing the nodal units of the cassava cuttings is one strategy to increase the availability of cassava planting materials for large-scale production (Remison et al., 2015; Moreno-Cadena et al., 2021). The leaves of the stems supply carbohydrates required for nodal unit maintenance and have an impact on root growth (Cock, 2011; Han et al., 2022). Due to the ability of cassava to multiply the planting material from a single node with leaf after 4 or 5 months of planting, the use of leaf bud cutting technique for cassava propagation becomes practical (Sheat et al., 2024). The leaf bud technique is among the rapid multiplication techniques of cassava (Neves et al., 2018). However, several factors influence the plantlet production through leaf buds, with the variety intended for propagation being a crucial consideration. Therefore, this study was conducted to assess how different cassava varieties and growth media respond to the leaf bud propagation technique for mass production of cassava planting materials.

## 2 Materials and methods

### 2.1 Study site description and duration

The experiment was conducted from November 2022 to February 2023 season at Tanzania Agricultural Research Institute (TARI) Tumbi Centre, Tabora Region, in the western part of Tanzania. The site is located between -5 04′ 0.01″ S and 32° 43′ 59.99″ E with an altitude of 1,190 m above sea level The area is characterized by an average temperature range of 22°C–31°C. The area receives a unimodal rainfall pattern of approximately 700 mm–800 mm per annum from November to April.

### 2.2 Planting materials

The cassava varieties, namely, Kizimbani, Mkombozi, Mkumba, TARICASS 4, and Mkuranga 1, which are the most commonly cultivated by farmers in the Western regions of Tanzania (Tabora, Katavi, and Kigoma), were selected for the study. The criteria for selection were tolerance to common cassava insect pests especially green mites (*Mononychellus tanajoa*); resistance to cassava mosaic disease (CMD), and cassava brown streak disease (CBSD), and high yielding, high dry matter, and starch content potential.

### 2.3 Media preparation

Peat moss and coco peat growth media were obtained from Balton (Tanzania) Limited. Coco peat, a by-product of the coconut (*Cocos nucifera L.*), is an important soilless media that contains high potassium (K), sodium (Na), and electrical conductivity (EC). Coco peat was soaked in tap water for 3 days, then buffered with Nitrabor fertilizer containing calcium using a ratio of 10 L of water: 20 g of CaO: 60 kg of Coco peat to reduce electro conductivity (EC) to a recommended value of 0.01 Ms/cm ideal for sprouting, root initiation, and plant growth, leaf bud plantlets for this subject matter. It was subsequently rinsed with water to adjust the pH to 7.5. EC and pH were measured using an EC and pH tester (Combo pH & EC tester HI 98130 HANNA, Mauritius). Peat moss was soaked in water for 30 min, to allow enough moisture accumulation ideal for sprouting and root induction condition. Washed white sand was obtained at the periphery of water catchment and steam sterilized to eliminate soil-borne pathogens before being used as third medium in the experiment.

### 2.4 Preparation and planting of leaf buds

Leaf buds were obtained from stem cuttings from branches of mature mother plants of each variety under investigation. The cuttings were collected early in the morning to ensure good turgidity. Leaves attached to buds were cut to one-third of the original size using alcohol-sterilized scissors and then kept in water to avoid leaf wilting. Leaf buds were removed from the branches in a “V”-cut fashion using a sterilized scalpel and then planted in plastic cups of 250 cm^3^ filled with three different media, namely, sand, coco peat, and peat moss. Cages made of metal pipes measuring 3.0 m × 1.5 m × 3.5 m, covered with transparent polyethylene sheet, were used for sprouting of leaf buds planted. The leaf buds were maintained in the growth cages for 30 days at a relative humidity of 85 ± 5% and temperature 30°C ± 2°C measured using a Big Digit Hygro-Thermometer (Extech Instruments, USA). The relative humidity and temperature were monitored by misting two to five times a day depending on the weather for 30 days and then transferred to the screen house for further growth, after which they were transferred to the screen house and managed for 60 days whereby they were mature enough and suitable for field establishment.

A split–split plot experiment was laid out in a randomized block design with three replications established. The main plot factor was cassava varieties (Kizimbani, Mkombozi, Mkumba, TARICASS 4, and Mkuranga 1), media (sterilized sand, coco peat, and peat moss) as a subplot factor, and leaf bud position (upper, middle bud, and lower bud position).

### 2.5 Data collection and analysis

Cassava planting materials were used in quantification of key parameters including the number of nodes attached with a leaf taken by counting from each of the three positions before excised for planting; days to first sprouting; the number of days taken for the first sprout to appear; the percentage of successful sprouting which is the proportion of leaf buds planted over the number of leaf bud sprouted; days to 50% sprouting determined by counting the average number of days taken for a half number of leaf buds to sprout and the number of sprouts per plot determined by counting the total number of sprouted leaf buds of a whole plot; and survival rate of sprouts of leaf bud plantlets determined by the proportion of plantlets survived over the total number of sprouts.

Analysis of variance was performed using GenStat statistical software 16^th^ edition (VSN international) for all parameters subjected to the study. Mean separation was done using a specific statistical model for split–split plot arrangement by identifying all possible sources of variation using Tukey’s honest significance test, at 5% significance (Payne et al., 2011).

## 3 Results

### 3.1 Number of leaf bud nodes

**Figure 1:**
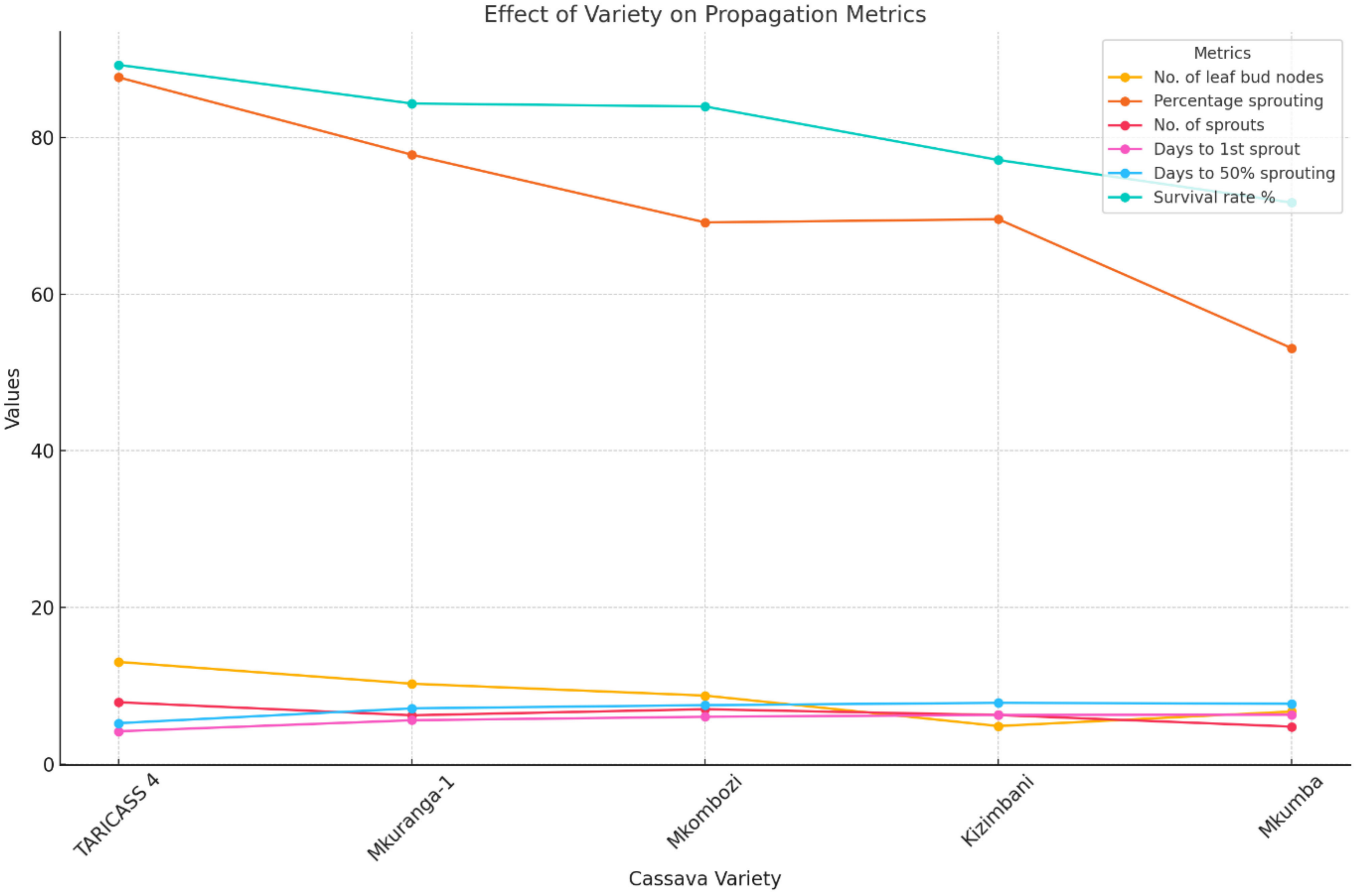
Pivot chart visualizing the effect of cassava varieties on the propagation metrics. Each line represents a different metric, showing the performance of each variety across all evaluated parameters.

The results showed that the number of leaf bud nodes was significantly (p < 0.001) affected by variety and leaf position (**Table 1**). For the variety type, TARICASS 4, Mkuranga 1, and Mkombozi had the higher average number of leaf bud nodes, 13, 10, and 8, respectively (**Figure 1**). The low numbers of leaf bud nodes were for Mkumba (6) and Kizimbani (4) (**Table 2**). Regarding the position of leaf buds, the upper leaf position had a higher average number of leaf buds (10) whereas the middle and lower positions had approximately the same number of leaf buds (8) in average (**Table 3**).

**Table 1:** Analysis of variance for number of leaf bud nodes, percent sprouting, number of sprout, days to first sprout, days to 50% sprouting, and survival rate of plantlets propagated from leaf buds.

Source of variation	DF	No. of leaf bud nodes	% sprout	No. of sprouts	Days to first sprout	Days to 50% sprouting	Survival rate %
Variety	4	270.23***	4,379.5 ***	35.47***	20.82**	30.87***	1,279.7***
Media	2	4.05ns	313.7ns	2.54ns	3.12**	4.83**	113.9ns
Variety*Media	8	0.7ns	203.9s	1.6519ns	0.6ns	1.3759ns	2.181**
Leaf bud position (LBP)	2	71.91***	851.4***	6.89***	8.14***	28.83***	3760.4***
Varity × LBP	8	3.34ns	189.5ns	1.54ns	0.21ns	0.26ns	33.9ns
Media × LBP	4	1.07ns	261.5**	2.11**	0.12ns	0.25ns	46.3ns
Variety × media × LBP	16	1.28ns	131.2ns	1.06ns	0.23ns	0.39ns	56.9ns
Coeffic. of var (CV%)		22.1	14.2	14.2	11.5	10.8	14.3

**, ***Significant at 1% and 0.1%, respectively, of probability by F test. ns, non-significant; LBP, leaf bud position; DF, degrees of freedom

**Table 2:** Effect of variety on number of leaf bud nodes, percent sprouting, number of sprout, days to first sprout, days to 50% sprouting, and survival rate of plantlets propagated from leaf buds.

Variety	No. of leaf bud nodes	Percentage sprouting	No. of sprouts	Days to first sprout	Days to 50% sprouting	Survival rate %
TARICASS 4	13.037 e	87.65 d	7.889 d	4.185 a	5.222 a	89.25 c
Mkuranga-1	10.259 d	77.78 c	6.222 b	5.593 ab	7.111 ab	84.32 bc
Mkombozi	8.741 c	69.14 b	7.000 c	6.037 b	7.519 b	83.94 bc
Kizimbani	4.852 a	69.55 bc	6.259 bc	6.259 b	7.815 b	77.11 ab
Mkumba	6.704 b	53.09 a	4.778 a	6.296 b	7.704 b	71.67 a
*P value*	0.001	0.001	0.001	0.01	0.001	0.001

Means followed by the same letter in the column of each parameter do not differ significantly from each other at p ≥ 0.05 according to Tukey’s HSD test.

**Table 3:** Effects of media and leaf bud position on the number of cassava sprouts and percent sprouting.

Trait	Media	Leaf bud position			
		Low	Middle	Upper	*P value*
No. of sprouts	Coco peat	6 ab	6.333 a	7.333 bc	0.01
	Peat moss	6.267 ab	6.467 a	6.667 bc	
	Sand	5.367	6.133 a	6.6 a	
% sprouting	Coco peat	74.07 ab	70.37 ab	81.48 c	0.01
	Peat moss	73.33 a	71.85ab	74.07 b	
	Sand	62.96 a	68.15 a	66.67 ab	

Means followed by the same letter in the column of each parameter do not differ significantly from each other at p ≥ 0.05 according to Tukey’s HSD test.

### 3.2 Percent sprouting

The results showed that the percentage sprouting was significantly affected by variety type (p<0.001), leaf bud position (p<0.001), and the interaction between media and leaf bud position (p < 0.01) (**Table 1**). The results indicated that the interaction between growth media and leaf bud position impacted the sprouting of cassava leaf buds, regardless of the variety. For the interaction between the variables, the results showed that the highest sprouting percentage was observed in the interaction between the of the two components, coco peat media and upper leaf buds, which gave a total of 81.48%, and the lowest sprouting percentage was observed in combination of sand and lower leaf bud position with 62.96% (**Table 3**). Concerning the variety type, TARICASS 4 variety had the highest sprouting percentage (87.65%), followed by Mkuranga with 77.78% (**Figure 1**). Mkombozi and Kizimbani had approximately the same sprouting percentage, 69.14% and 69.55%, respectively. The lowest sprouting percentage (53.09%) was observed for the variety Kizimbani (**Table 2**). For the leaf bud position variable, leaf buds from the upper position had a higher sprouting percentage (76.3%) compared with those from the middle and lower positions with 70.12% and 67.9%, respectively, regardless of the variety and media (**Table 4**).

**Table 4:** Effects of leaf bud position on number of leaf bud nodes, percent sprouting, number of sprout, days to first sprout, days to 50% sprouting, and survival rate of plantlets propagated from leaf buds.

Leaf bud position	No. of leaf buds per branch	% sprouting	No. of sprouts	Days to first sprout	Days to 50% sprouting	Survival rate %
Upper	10.18 c	76.3 a	6.867 c	5.222 a	6.289 a	89.9 c
Middle	8.02 b	70.12 ab	6.311 ab	5.733 b	7.044 b	82.19 b
Lower	7.96 a	67.9 c	6.111 a	6.067 c	7.889 c	71.69 a
P-value	0.001	0.001	0.001	0.001	0.001	0.001

Means followed by the same letter in the column of each parameter do not differ significantly from each other at p ≥ 0.05 according to Tukey’s HSD test.

### 3.3 Number of sprouts

The results showed that the number of sprouts was significantly influenced by variety (p < 0.001) and leaf bud position (p < 0.001). Furthermore, the interaction between media and leaf bud position was significant at p < 0.05 (**Table 1**). Looking at the interaction, a large number of sprouts was obtained in a combination of coco peat media and upper leaf buds, which gave rise to an average of seven (7) plantlets, whereas the lowest number of sprout, five in average, was found in a combination of sand leaf buds with 5 number of plantlets in average (**Table 3**). For the performance of varieties, TARICASS 4 and Mkombozi had many number of sprouts per plot, 7 and 8 numbers of sprouts in average, followed by Mkuranga 1 and Kizimbani with 6 number of sprouts (**Figure 1**). The lowest number of sprouts, 4 in average, was observed in the Mkumba variety (**Table 2**). Furthermore, number of sprouts was also affected by the leaf bud position whereby the upper position had a higher number of sprouts 7 in average. The middle and lower positions had approximately the same number of sprouts, 6 in average (**Table 4**).

### 3.4 Days to first sprout

Variety type and growth media showed a significant (p < 0.01) effect on the days to the first sprout. It was further found that the leaf bud position had a significant (p < 0.001) effect on the days to first sprout (**Table 1**). The interaction between variables was not significant, suggesting that the combined effect of the used treatment had no impact on days on first sprouting of cassava leaf buds. Among all five varieties, TARICASS 4 sprouted in 4 days, indicating earlier sprouting potential, followed by variety Mkuranga which sprouted in average of 5 days (**Figure 1**). The other three varieties, Mkombozi, Kizimbani, and Mkumba, delayed for approximately 6 days to give out the first sprout (**Table 2**). For the three leaf bud positions subjected to the study, the upper leaf buds took 5 days in average to give the first sprout which was observed as the earlier sprouting compared with middle and lower leaf buds, which took an average of 6 days to give the first sprout (**Table 4**).

**SCHEME 1:**
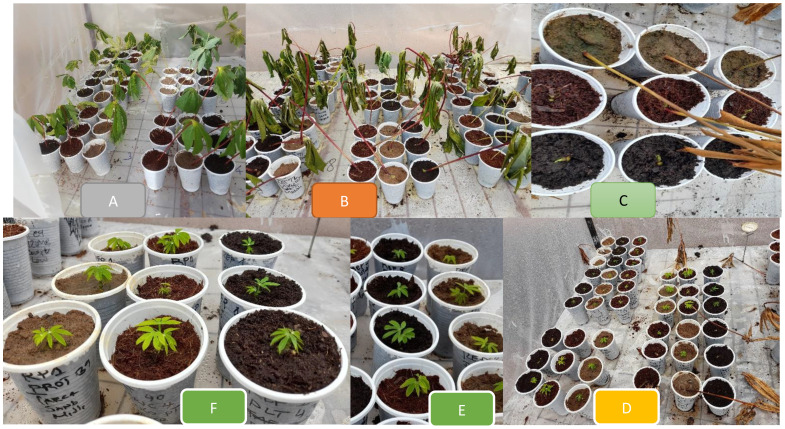
Cassava leaf bud photos showing the experimental growing stages. **(A)** Leaf bud planted in the plastic cups with different growth media. **(B)** Leaves attached with buds started wilting 3 days after planting. **(C)** Leaf buds started sprouting 4 to 5 days after planting. **(D)** Leaf bud plantlets growth 14 days after planting **(E, F)** Leaf bud plantlets growth 21 days after planting

### 3.5 Days to 50% sprouting

The results showed that variety type and leaf bud position had a significant (p < 0.001) effect on the 50% sprouting. The effects of growth media used to anchor leaf buds for sprouting was also significant (p < 0.01) and influenced the days to 50% sprouting of cassava plantlets (**Table 1**. Regarding the variety type, TARICASS 4 reached 50% sprouting earlier than the other varieties regardless of the leaf bud position with an average of 5 days to 50% sprouting, whereas the other four varieties, namely, Mkuranga 1, Mkombozi, Kizimbani, and Mkumba, took approximately 7 days to 50% sprouting (**Table 2**). Relying on the position of leaf buds, leaf buds excised from the upper position achieved 50% sprouting earlier with an average of 6 days, followed by leaf buds from the middle position with 7 days to 50% sprouting. Leaf buds from the lower position took long time to reach 50% sprouting, 8 days in average (**Table 4**).

### 3.6 Survival rate

The results showed that there was a significant difference among the varieties (p < 0.001) and position of leaf buds < 0.001 (**Table 1**). Varietal component impacted the percent at which the plantlets survived, whereby there was a significant variation among all the five varieties. For the varieties, TARICASS 4 plantlets had the highest survival rate (89.25%) tailed by Mkuranga 1 (84.32%) and Mkombozi (83.94%) with closely the same survival rate of their plantlets (**Figure 1**). The lower survival rate was shown by Kizimbani (77.11) and Mkumba (71.67%), respectively (**Table 2**). Also, regardless of the media used, leaf bud plantlets from the lower position showed a higher survival rate (89.9%) in comparison with those plantlets derived from middle (82.19) and lower position (71.69%) correspondingly (**Table 4**).

## 4 Discussion

Recently, the adoption of improved cassava varieties by farmers has continuously increased due to the shift of cassava production from subsistence to a commercial crop. However, the area under cultivation is still low due to low availability of planting materials especially for the newly released improved varieties (Mtunguja et al., 2019). The low planting material availability of improved varieties to farmers is due to the poor multiplication rate of cassava through a conventional propagation method and unavailability of rapid techniques for mass propagation of planting materials to farmers. Using the leaf bud technique for seed increase in both breeding and seed system programs can boost the efforts being made to increase the availability and use of improved cassava varieties by farmers (Neves et al., 2019). Also, identification of cassava varieties with a good response to the technique can provide sustainable and inexpensive opportunities to multiply the planting materials, ranging from the breeder seed to certified seeds of the released varieties, and easy accessibility to farmers. This study focused on the assessment of the response of improved cassava varieties for use in the leaf bud technique, the position where the leaf bud excised, and the media used for sprouting of the leaf buds. In general, this study revealed that significant differences in response of the varieties used on leaf bud sprouting send their subsequent phenotypic traits assessed. This differential response in sprouting is typically due to genotypic differences of the varieties (Ceballos et al., 2020), since the leaf bud plantlets obtained from the same branch position, raised using the same media and on same environmental conditions, presented different values in days to first sprouting, number of sprouts, and days to 50% sprouting. The difference in the response of the varieties to sprouting of their leaf buds are in conformity with findings by Neves et al. (2019) who reported that one of the factors to be taken into consideration in the use of leaf bud technique is the variety to be propagated as they differ in response due to difference in maturation of the stem resulting to differences in rooting and sprouting of buds.

From the findings of this study, the TARICASS 4 variety had outstanding performance by having a higher response to leaf bud technique including the average number of days (4) to give out the first sprout, whereas other varieties took 5 to 6 days. in addition, TARICASS 4 had the highest percentage of sprouting, regardless of the position and medium used followed by Mkuranga 1. For the Mkombozi variety, the sprouting percentage was good, but its leaf buds took an average of 6 days to give rise to the first sprout. Among all the five varieties, Mkumba had a poor response to all traits assessed, making it unsuitable for propagation using leaf buds. The positive results demonstrated by TARICASS 4 outperforming the other varieties across all the assessed traits suggest its higher potential and leaf bud technique a choice for its propagation.

The study also utilized three different growth media for the sprouting of cassava leaf buds: coco peat, peat moss, and sand. Coco peat and peat moss led to a desirable effect on the sprouting of the leaf buds regardless of the variety type and position from which the leaf buds were excised. Leaf buds raised from coco peat media had a percentage sprouting of 73.09%, and those raised in peat moss had a sprouting percentage of 72.84%. This is due to the ability of the two media to hold water and hence retain enough moisture ideal for sprouting of leaf buds. This also resulted to shorter time to sprouting, whereby leaf buds planted in coco peat media took an average of 5 days to give the first sprout, unlike sand with a poor water holding capacity resulting from a lower sprouting percentage (68.4%) of the leaf buds raised and also resulting to longer time to sprouting (6 days on average).

The position of leaf buds on the mother plant branch was found as another significant factor to consider. Considering the percentage sprouting, leaf buds from the upper branch position showed the highest sprouting percentage (76.3%) compared with the middle (70.12%) and lower (67.9%) positions. Regarding days to first sprouting, leaf buds from the upper position sprouted earliest (5 days on average) compared with the middle and lower positions both with 6 days to first sprouting. With respect to days to 50% sprouting, leaf buds from the upper position also sprouted earlier (6 days on average) compared with the middle and lower positions (7 days on average). The highest sprout percentage, earliest sprouting of cassava leaf buds, was obtained from the upper position which can be attributed to the herbaceous nature of apical plant parts (Alves, 2001; Muktar et al., 2024). This led to an increase in sprout percentage and shorter sprouting duration due to the lower degree of tissue lignification in the herbaceous position where the leaf buds were obtained. These findings are in conformity with those observed by Neves et al. (2019) and Oruonye et al. (2021), who found that apical cuttings, with a lower degree of lignification, conferred supreme physiological conditions such as the presence of auxins and vital substances for greater rooting and sprouting of plantlets from leaf buds of *Lavandula dentata* L. These findings suggest that the upper position of leaf buds is preferable for cassava propagation due to higher sprouting percentages and shorter sprouting duration.

With respect to the interaction between variety and media, it was found that regardless of the media used, there was a significant difference in days to first sprouting. TARICASS 4 consistently led to earlier sprouting across all media. In the case of percentage sprouting, there was no significant interaction between media and variety. For the survival rate, plantlets propagated from the upper leaf buds had a higher survival rate than those from the middle and lower positions due to the initial earlier sprouting, which led to shorter time to establish roots for water uptake and leaves for food synthesis through photosynthesis, unlike the leaf buds from the middle and lower positions which delayed in their initial establishment and thus struggled in their survival. The research also looked at the interaction between the position of leaf buds and media. There was a significant interaction between media and leaf bud position on the number of sprouts and percentage sprouting, indicating that the combination of coco peat and leaf buds from the upper position had a higher sprouting percentage and large number of sprouts, indicating their favorability to leaf bud propagation technique. For days to first sprouting and survival rate, media had a more pronounced effect than the position of leaf buds. Similar findings were obtained by Neves et al. (2019), stating that cassava plantlet propagation by leaf buds revealed differential responses, because the duration of the stem maturation varies regarding the genetic difference of the varieties used. Our current study revealed that different cassava varieties planted under the same conditions presented different levels of development and physiological maturation. The case of TARICASS 4 showed an outstanding performance for all traits assessed ranging from number of sprouts to survival rate of plantlets. These findings are valuable for optimizing cassava leaf bud propagation, helping to select the right varieties, growth media, and leaf bud positions to maximize the efficiency and success of cassava planting material production.

## 5 Conclusion and recommendation

### 5.1 Conclusion

The study has provided valuable insights into the factors affecting the sprouting of cassava leaf bud plantlets. The results showed that the choice of variety, media, and leaf bud position significantly influence the sprouting of cassava leaf bud plantlets. TARICASS 4 was the most promising variety for rapid multiplication of cassava planting materials using the leaf bud technique due to its superior performance. The use of coco peat and peat moss media generally leads to early and more successful sprouting compared with sand. Leaf bud position, specifically the upper position, was found to be more favorable for sprouting, indicating that leaf buds from this branch position are the most suitable for propagation. The interaction between media and leaf bud position highlights the importance of selecting the right combination to optimize sprouting. This could have practical implications for cassava propagation strategies for both research and mass propagation of planting materials for farmers.

### 5.2 Recommendations

Based on our current findings, TARICASS 4 is recommended as a variety for cassava propagation using the leaf bud technique for rapid multiplication of cassava planting materials. Coco peat and peat moss are recommended as the preferred media for cassava leaf bud sprouting since they lead to faster and more successful sprouting results. Consider using leaf buds from the upper position on cassava plants to maximize sprouting efficiency. Practitioners in cassava farming should take these findings into account when selecting planting materials and propagation methods to enhance cassava production.

Research needs to be extended to explore at the molecular level to identify the gene responsible for sprouting of leaf buds, and TARICASS 4 variety can be one among the sources of genes. Identifying the genes responsible for sprouting in cassava will help to develop varieties with high sprouting ability and become a sustainable means for multiplication of cassava materials in both breeding and seed system programs.

## Data Availability

The original contribution presented in the study are included in the article, further inquiries can be directed to the corresponding author.
